# A process to deduplicate individuals for regional chronic disease prevalence estimates using a distributed data network of electronic health records

**DOI:** 10.1002/lrh2.10297

**Published:** 2021-11-28

**Authors:** Kenneth A. Scott, Sara Deakyne Davies, Rachel Zucker, Toan Ong, Emily McCormick Kraus, Michael G Kahn, Jessica Bondy, Matt F. Daley, Kate Horle, Emily Bacon, Lisa Schilling, Tessa Crume, Romana Hasnain‐Wynia, Seth Foldy, Gregory Budney, Arthur J. Davidson

**Affiliations:** ^1^ Denver Public Health Denver Health Denver Colorado USA; ^2^ Department of Epidemiology Colorado School of Public Health, University of Colorado Anschutz Medical Campus Aurora Colorado USA; ^3^ Analytics Research Center Children's Hospital Colorado Aurora Colorado USA; ^4^ Adult and Child Consortium for Health Outcomes Research and Delivery Science (ACCORDS) University of Colorado Anschutz Medical Campus Aurora Colorado USA; ^5^ Department of Pediatrics School of Medicine, University of Colorado Anschutz Medical Campus Aurora Colorado USA; ^6^ Kraushold Consulting LLC Atlanta Georgia USA; ^7^ Department of Biostatistics and Informatics Colorado School of Public Health, University of Colorado Anschutz Medical Campus Denver Colorado USA; ^8^ Bacon Analytics, LLC Denver Colorado USA; ^9^ Institute for Health Research, Kaiser Permanente Colorado Aurora Colorado USA; ^10^ CORHIO Denver Colorado USA; ^11^ Division of General Internal Medicine, Department of Medicine University of Colorado Denver School of Medicine Aurora Colorado USA; ^12^ Office of Research Denver Health Denver Colorado USA

**Keywords:** electronic health records, medical record linkage, network, public health informatics, public health surveillance

## Abstract

**Introduction:**

Learning health systems can help estimate chronic disease prevalence through distributed data networks (DDNs). Concerns remain about bias introduced to DDN prevalence estimates when individuals seeking care across systems are counted multiple times. This paper describes a process to deduplicate individuals for DDN prevalence estimates.

**Methods:**

We operationalized a two‐step deduplication process, leveraging health information exchange (HIE)‐assigned network identifiers, within the Colorado Health Observation Regional Data Service (CHORDS) DDN. We generated prevalence estimates for type 1 and type 2 diabetes among pediatric patients (0‐17 years) with at least one 2017 encounter in one of two geographically‐proximate DDN partners. We assessed the extent of cross‐system duplication and its effect on prevalence estimates.

**Results:**

We identified 218 437 unique pediatric patients seen across systems during 2017, including 7628 (3.5%) seen in both. We found no measurable difference in prevalence after deduplication. The number of cases we identified differed slightly by data reconciliation strategy. Concordance of linked patients' demographic attributes varied by attribute.

**Conclusions:**

We implemented an HIE‐dependent, extensible process that deduplicates individuals for less biased prevalence estimates in a DDN. Our null pilot findings have limited generalizability. Overlap was small and likely insufficient to influence prevalence estimates. Other factors, including the number and size of partners, the matching algorithm, and the electronic phenotype may influence the degree of deduplication bias. Additional use cases may help improve understanding of duplication bias and reveal other principles and insights. This study informed how DDNs could support learning health systems' response to public health challenges and improve regional health.

## 1 INTRODUCTION

Learning health systems (LHSs) that leverage data for rapid, continuous improvement operate amid broader secular epidemics of chronic disease and substance use that exceed any one healthcare system's ability to address.^1^, ^2^, ^3^, ^4^ Public health problems freely transcend county boundaries and provider networks. A nationwide LHS, based on a federated data sharing model,^5^ proposes to combine LHS concepts with established public health strategies, such as estimating disease prevalence.^6^ One common LHS challenge is reconciling fragmented data collected across an ecosystem of electronic health records (EHRs). Record fragmentation limits a single system's ability to learn from patients' experiences and outcomes at the individual or population level. A treatment (or exposure) may be recorded in one healthcare system, while related outcomes may be recorded in another. Conceptually, this identity management (IM) challenge includes several component activities: (a) uniquely identifying and linking individuals across multiple data sources and distinct healthcare organizations, (b) aggregating individual‐level health data from multiple sources, and (c) reconciling data and discrepancies across sources (eg, removing duplicates, resolving changing residence data over time). For example, while healthcare organizations identify unique individuals and assign medical record numbers using internal IM tools, health information exchanges (HIEs) facilitate Health Insurance Portability and Accountability Act‐(HIPAA) compliant, data sharing from one covered entity to another using cross‐entity IM (eg, master patient index).^7^, ^8^

EHR distributed data networks (DDNs) can leverage federal investments for research, quality improvement and public health, valued domains for any LHS. Federated data sharing, recognized by funding agencies,^9^ and adopted by clinical data research networks,^10^, ^11^ can preserve privacy and security as data remain behind firewalls of DDN‐participating healthcare organizations, until queried for specific approved uses. The importance of IM in a DDN is likely influenced by the specific use case and geographic proximity of participating organizations. For example, whereas some PCORnet clinical data research networks may have limited geographic overlap and duplication of patients, others have implemented IM solutions.^12^, ^13^ Regional DDNs designed for quality improvement or public health surveillance in a defined region may be especially likely to experience patient duplication. Risks of duplication bias in public health DDNs have been recognized but lack data to inform decisions.^14^ Prevalence estimates from DDNs can be biased when individuals access multiple health systems are represented more than once,^15^ however the degree of bias may differ by use case.

Efforts to define, scope, and address problems caused by duplication for a variety of public health use cases are needed.^16^ Building on previous DDN‐based surveillance^17^, ^18^, ^19^ we sought to implement and evaluate methods to deduplicate DDN prevalence estimates. For pilot use cases we selected T1DM and T2DM, health conditions that require enhanced coordination across primary and specialty care settings. Across settings multiple records may exist for the same child, leading to potentially biased prevalence estimates. Our goal was to empirically evaluate a scalable process to deduplicate T1DM or T2DM prevalence estimates among pediatric patients receiving healthcare services at two large health systems in the same region.

## 2 METHODS

### 2.1 Setting

The Denver metropolitan area, an urban and suburban region, has collaboratively developed a DDN (ie, Colorado Health Observation Regional Data Service [CHORDS]) through a consortium of state/local public health departments, health systems, federally qualified health centers (FQHCs), community mental health centers, a regional HIE, a university, non‐profit organizations, and other key stakeholders. Data from EHRs are normalized to a common data model and queried using DDN data aggregation software (ie, PopMedNet [PMN]).^20^ A detailed description of the development of CHORDS has been published elsewhere.^17^ This study focuses on an IM approach that could scale to deduplicating prevalence estimates in the seven‐county Denver metropolitan area, which includes over 50% of Colorado's 5.8 million residents.

### 2.2 Populations

Two health systems contributing data to the CHORDS Network (“data partners”) participated in this study. Data partner 1 (DP1) is a large, integrated safety‐net health system recognized as an LHS^9^ that provides care for the majority of low‐income individuals in the City and County of Denver (~30% of Denver's population). Data partner 2 (DP2) is a large pediatric tertiary care facility that participates in a network LHS.^10^ Children seen in primary care at DP1 are routinely referred to DP2 for many types of specialty care. Patients with T1DM are referred to a specialty diabetes program affiliated with DP2 that did not contribute data to this study. Both data partners have multiple locations throughout the Denver metropolitan area. DP1 and DP2 operate locations as close as 3 miles of one another.

**FIGURE 1:**
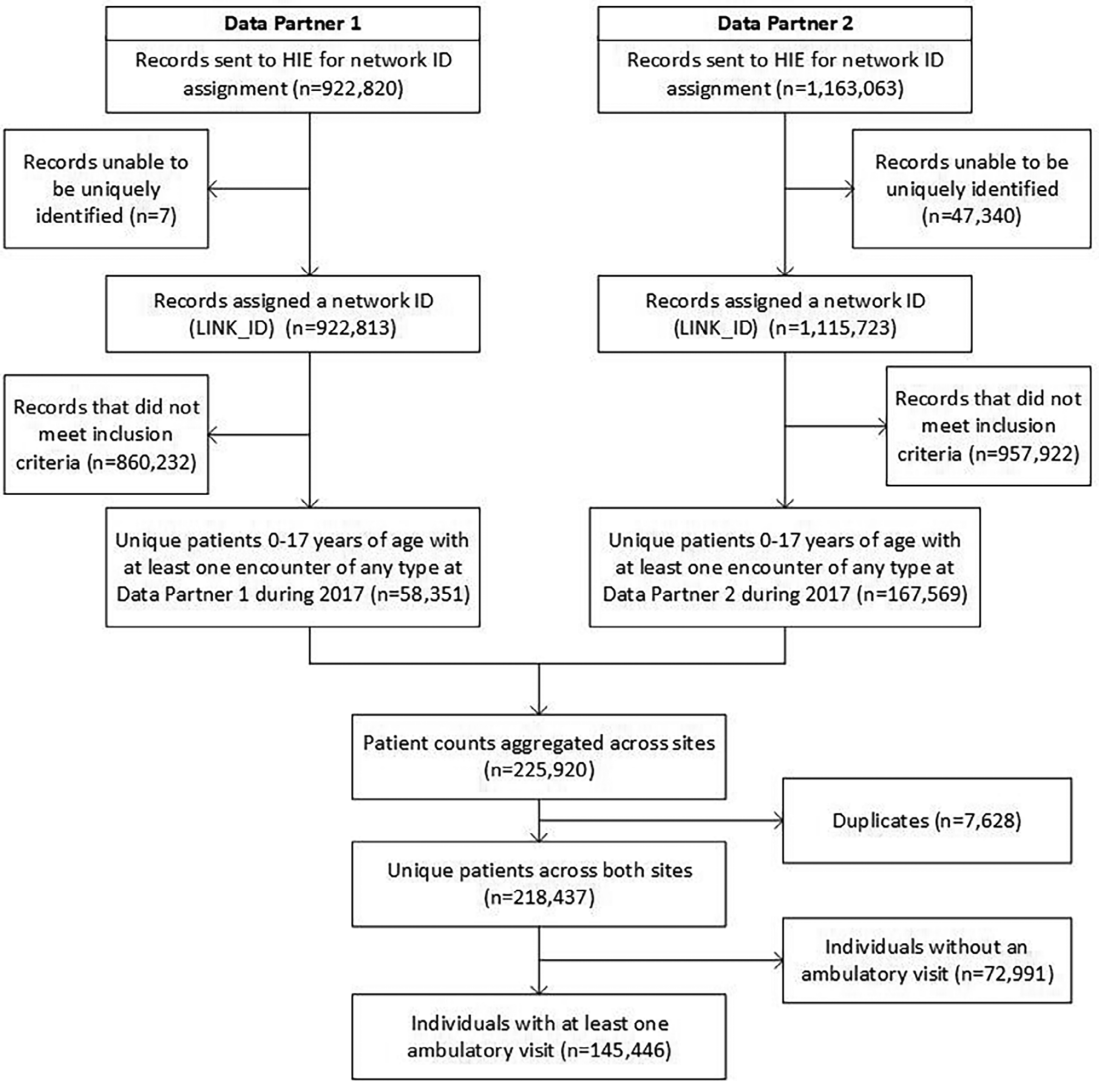
Strobe flow diagram representing the number of unique patients across two distributed data network partners participating in a study of identity management's influence of type 1 and type 2 diabetes prevalence

The eligible population (denominator) for this evaluation were children (less than 18 years of age on the date of the encounter) with at least one 2017 healthcare encounter at either data partner, residing in the seven‐county Denver metropolitan. Individuals with incomplete information for unique identification by the HIE were excluded (n = 47 347, 2% of all records; see Figure 1).

For its chronic disease surveillance mission the CHORDS Network leverages numerous case definitions drawn from the Centers for Medicare and Medicaid Services Chronic Conditions Data Warehouse.^21^ Cases were identified individuals with at least one International Classification of Disease (ICD) code for a billing or problem list diagnosis of T1DM or T2DM. Individuals with at least one T1DM diagnosis, at either data partner, were classified as T1DM cases. Likewise, a single T2DM diagnosis resulted in a person being classified as a T2DM case. We did not distinguish cases with both T1DM and T2DM diagnosis codes.

### 2.3 Data sources and distributed network

*Data Governance*: Data partners executed a Data Use Agreement (DUA) to share a record‐level limited dataset and business associate agreements (BAA) to share personally identifiable information (PII) with the HIE. The HIE has participation agreements with each data partner and routinely manages patients' identities as part of its core business functions. The HIE assigned a unique network‐wide identifier (ie, LINK_ID) for each patient, which linked patients across data partners. The Colorado Multiple Institutional Review Board reviewed the CHORDS Network as non‐human subjects research for public health uses.

**FIGURE 2:**
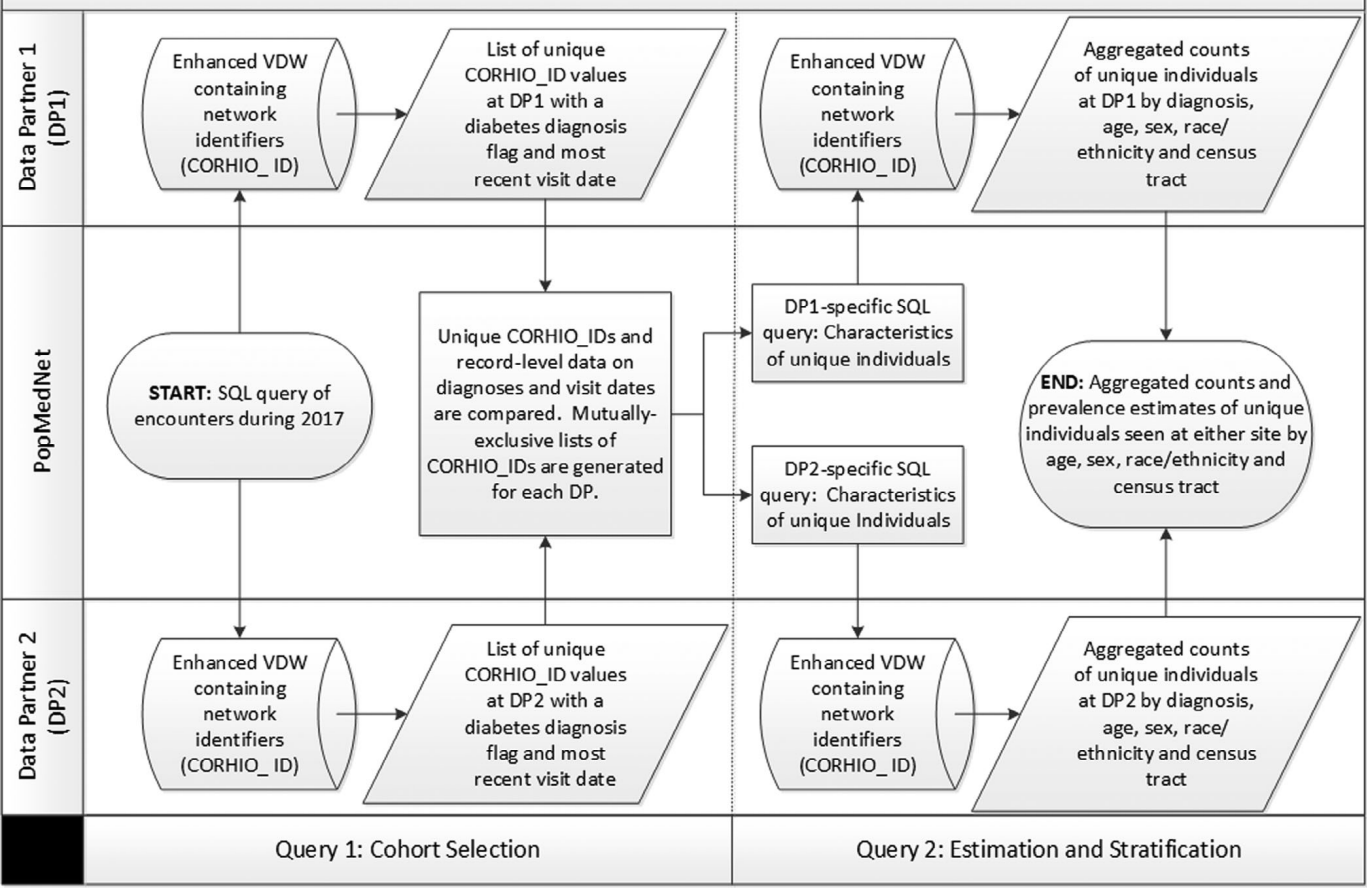
Generating stratified, deduplicated estimates of diabetes prevalence through a distributed query process that minimizes exchange of phi

*Patient Matching*: Data partners generated a panel file containing pertinent demographic data for every individual seen from their data warehouse (Figure 2). Each record in the panel file included a site‐specific identifier (PERSON_ID) and a series of pre‐specified PII fields. Data partners transmitted panel files to/from the HIE using secure file transfer protocols (SFTP). The HIE used a proprietary referential matching process that combined a database of PII, with rules‐based, probabilistic linkage methods to identify unique individuals in the panel file and assign a unique, network‐wide identifier for linkage (LINK_ID). The HIE returned the LINK_ID and PERSON_ID for each patient back to each data partner.

*CHORDS Data Model*: CHORDS used a data model (ie, Virtual Data Warehouse) adapted from other common data models.^22^, ^23^ Both data partners extracted, transformed, and loaded (ETL) EHR data into 24 distinct tables, with specific fields and data formats organized for PMN queries. Network identifiers were stored in the LINKAGE table (Figure 2), where one or more rows were associated with each LINK_ID. More than one row was required when the patient matching algorithm identified duplicates within a data partner and assigned the same network‐wide identifier (LINK_ID) to several different individuals (eg, PERSON_ID).

*Distributed Query Logic*: We developed a two‐step query process: (a) select a cohort of unique individuals across data partners and (b) classify those individuals into cases (yes/no). Limited exchange of associated PII permitted demographic (age, gender, and race/ethnicity) and geographic (census tract) stratification. We designed an automated process to reconcile medical, demographic, and geographic information that conflicted across partners (eg, an individual may be diagnosed at one data partner and not another). Manual reconciliation was resource‐intensive and infeasible. Automated reconciliation required decisions to identify which value(s) to use in estimating prevalence. Individual‐level data exchange was limited to three variables: LINK_ID, diagnosis status and final 2017 visit date. Individual demographic and geographic data were selected from the system whose data were used in Step 2. Below, we describe the two‐step (ie, cohort selection and stratification) query processes:

*Cohort Reconciliation*: For each condition (T1DM or T2DM) a cohort was selected by generating lists of all eligible individuals (see above) with an initial query. Query results contained three fields: the LINK_ID, a binary indicator for case status and last visit date. With these fields a single data partner was selected to contribute a given patient's data (medical, demographic, geographic) to prevalence estimates. Rules for data partner selection are described below (mock results are represented in Table 1).

- *Decision 1*: When an individual (CID1) was seen by both data partners, yet only one identified the patient as a case, select the record from the data partner identifying the patient as a case.
- *Decision 2*: When an individual (CID2 or CID3) was seen by both data partners, and has the same case status, select the record from the data partner with the most recent 2017 visit.
- *Decision 3*: When an individual (CID4 or CID5) was seen by both data partners, and has the same case status and the same most recent 2017 visit date, select the data partner at random.

**TABLE 1:** Example of reconciliation process for selecting data partners to contribute demographic and geographic data for individuals seen in multiple health care systems (selection criteria are highlighted)

Network identifier	Diagnosis present		Final 2017 visit date		Selected data partner
	Date partner		Data partner		
	1	2	1	2	
CID1	Yes	No	January 1, 2017	December 31, 2017	1
CID2	Yes	Yes	December 31, 2017	January 1, 2017	1
CID3	No	No	January 1, 2017	December 31, 2017	2
CID4	Yes	Yes	January 1, 2017	January 1, 2017	2 (random)
CID5	No	No	December 31, 2017	December 31, 2017	1 (random)

The query tool applied these logical rules to produce two mutually‐exclusive lists of LINK_IDs ‐ one for each data partner.

*Stratification*: The second query produced aggregate counts, limited to the lists of patients identified through reconciliation. Each data partner's care population and cases were grouped by demographic and geographic factors. American Community Survey data for neighborhood poverty (ie, greater than 20% of population living below the federal poverty level: yes/no) was assigned for each patient, based on census tract of residence. We conducted additional analyses, limited to data on ambulatory encounters and following the same analytic approach, to assess the impact of care setting on prevalence estimates and on duplication bias. Once data partners incorporated the Network Identifier and ensured patient populations were distinct, each data partner returned tables of counts. Stratum‐specific counts were summed across data partners and used to generate prevalence estimates for the cohort overall and for each stratum.

### 2.4 Analytic approach

*Outcome*: The primary outcomes of interest were the prevalence of T1DM and T2DM in a pediatric population. Estimated prevalence before deduplication, within and between systems, was calculated by dividing total number of individuals with a diabetes diagnosis (of a given type) by the number of eligible patients. De‐duplicated prevalence estimates divided the unique number of cases by the unique number of eligible patients. We reported confidence intervals (95%) for all prevalence estimates. Because the process of selecting data for an individual may influence case counts and prevalence estimates we tested three alternative decision rules: selecting the data source with the latest 2017 encounter, while ignoring case status (Alternate 1); selecting the data source with the initial 2017 encounter, while ignoring case status (Alternate 2); and selecting the data partner at random (Alternate 3).

## 3 RESULTS

Among 58 351 eligible children seen at DP1 and 167 569 seen at DP2 (Table 2), the DP2 population had a higher T1DM prevalence and a lower T2DM prevalence compared to the DP1 population. DP2's population was younger, had a greater proportion of male and white patients, and a smaller proportion of patients of Hispanic ethnicity than the DP1 population.

**TABLE 2:** Distribution of demographic characteristics and disease prevalence for patient populations (<19 years old) with any encounter during the study period among two data partners, seven‐county Denver metro area, 2017

	Data partner		*P*‐value
	1	2	
Number of patients	58 351	167 569	n/a
Diabetes prevalence (per 1000)			
Type 1	1.6	4.1	<.0001
Type 2	1.2	0.9	.03
Sex (percent)			<.0001
Female	50	48	
Male	50	52	
Unknown	0	<1	
Age group in years (percent)			<.0001
0‐3	21	33	
4‐6	16	16	
7‐9	16	15	
10‐12	17	14	
13‐15	17	14	
16‐17	13	8	
Race and ethnicity (percent)			<.0001
Non‐Hispanic (NH) White	13	46	
Hispanic	68	32	
NH Black	14	8	
NH Asian	4	3	
NH American Indian or Alaska Native	<1	<1	
NH multiple races	1	4	
NH race unknown or not reported	2	7	
Residing in census tract with > = 20% below federal poverty level (percent)^a^			<.0001
Yes	44	18	
No	56	82	

*Note*: *P*‐values calculated using Pearson's Chi‐squared test.

^a^ Some addresses could not be geolocated to the census tract.

Aggregation across data partners, without deduplication, would have estimated 226 100 children from the Denver region seen by these two data partners. We identified 218 437 unique individuals after deduplication, with 7628 (3.5%) seen in both systems (Table 3). Individuals seen by both data partners had a higher prevalence of both T1DM and T2DM than individuals seen in a single system. Compared to individuals seen in only one system, duplicates were more likely to be identified as Hispanic, non‐Hispanic black, or non‐Hispanic Asian, and were substantially more likely to reside in a higher poverty neighborhood.

**TABLE 3:** Distribution of demographic characteristics and disease prevalence for patient populations (<19 years old) with any encounter during the study period among two data partners, by duplicate status, seven‐county Denver metro area, 2017

	Duplicate status		
	Yes	No	*P*‐value
Number of patients	7628	210 809	
Diabetes prevalence (per 1000)			
Type 1	5	3.4	.03
Type 2	4	0.8	<.0001
Sex (percent)			.13
Female	49	48	
Male	51	52	
Unknown	0	<1	
Age Group in Years (percent)			<.0001
0‐3	20	30	
4‐6	19	16	
7‐9	17	15	
10‐12	15	15	
13‐15	17	15	
16‐18	11	9	
Race and ethnicity (percent)			<.0001
Non‐Hispanic (NH) White	10%	40%	
Hispanic	64%	39%	
NH Black	16%	9%	
NH Asian	4%	3%	
NH American Indian or Alaska Native	<1%	<1%	
NH multiple races	1%	3%	
NH race unknown or not reported	3%	6%	
Residing in census tract with > = 20% below federal poverty level (percent)^a^			<.0001
Yes	46%	23%	
No	54%	77%	

^a^ Some addresses could not be geolocated to the census tract.

The prevalence estimates of T1DM and T2DM before and after deduplication are presented in Table 4. Prevalence did not change after IM processes for either condition. There was no observed change in prevalence for any demographic or geographic subgroup after deduplication, even for the subgroups that were most affected by deduplication (eg, Hispanic patients).

**TABLE 4:** Prevalence (per 1000) of Type 1 and Type 2 diabetes among patient populations (<19 years) for all encounter types from two health care systems, before and after deduplication, seven‐county Denver Metropolitan Area, Colorado, 2017

	Deduplication			
	Type 1		Type 2	
	Before	After	Before	After
Overall	3.4 (3.2, 3.6)	3.5 (3.3, 3.7)	1.0 (0.9, 1.1)	0.9 (0.8, 1.0)
Sex				
Female	3.6 (3.2, 4.0)	3.7 (3.3, 4.1)	1.1 (0.9, 1.3)	1.0 (0.8, 1.2)
Male	3.3 (3.0, 3.6)	3.3 (3.0, 3.6)	0.8 (0.6, 1.0)	0.8 (0.6, 1.0)
Age in years				
0‐3	0.4 (0.2, 0.6)	0.4 (0.2, 0.6)	0 (0, 0)	0 (0, 0)
4‐6	1.6 (1.2, 2.0)	1.6 (1.2, 2.0)	0 (0, 0)	0 (0, 0)
7‐9	3.2 (2.6, 3.8)	3.2 (2.6, 3.8)	0.3 (0.1, 0.5)	0.3 (0.1, 0.5)
10‐12	5.5 (4.7, 6.3)	5.6 (4.8, 6.4)	0.8 (0.5, 1.1)	0.8 (0.5, 1.1)
13‐15	7.1 (6.2, 8.0)	7.2 (6.3, 8.1)	2.2 (1.7, 2.7)	2.0 (1.5, 2.5)
16‐17	7.5 (6.3, 8.7)	7.7 (6.5, 8.9)	4.9 (4.0, 5.8)	4.8 (3.8, 5.8)
Race				
Non‐Hispanic (NH) White	5.5 (5.0, 6.0)	5.5 (5.0, 6.0)	0.5 (0.3, 0.7)	0.5 (0.3, 0.7)
Hispanic	1.9 (1.6, 2.2)	1.9 (1.6, 2.2)	1.4 (1.2, 1.6)	1.3 (1.1, 1.5)
NH Black	3.1 (2.3, 3.9)	3.0 (2.2, 3.8)	1.8 (1.2, 2.4)	1.6 (1.0, 2.2)
NH Asian	1.0 (0.3, 1.7)	1.1 (0.3, 1.9)	0.6 (0.0, 1.2)	0.6 (0.0, 1.2)
NH American Indian or Alaska Native	4.3 (−0.6, 9.2)	4.5 (−0.6, 9.6)	2.9 (−1.1, 6.9)	3.0 (−1.1, 7.2)
NH multiple races	2.5 (1.3, 3.7)	2.5 (1.3, 3.7)	0.4 (−0.1, 0.9)	0.4 (−0.1, 0.9)
NH race unknown or not reported	3.4 (2.4, 4.4)	3.4 (2.4, 4.4)	0.2 (0.0, 0.4)	0.2 (−0.1, 0.5)
Residing in census tract with > = 20% below federal poverty level^a^				
Yes	2.2 (1.8, 2.6)	2.1 (1.7, 2.5)	1.4 (1.1, 1.7)	1.3 (1.0, 1.6)
No	3.9 (3.6, 4.2)	3.9 (3.6, 4.2)	0.8 (0.7, 0.9)	0.8 (0.7, 0.9)

^a^ Some addresses could not be geolocated to the census tract.

Concordance of recorded demographic attributes for duplicate patients was variable. Duplicate patients had high recorded gender agreement (98%) and were likely to have the same case status (>99% for both T1DM and T2DM). However, substantial discordance of recorded race and ethnicity was observed between systems. Agreement of Hispanic ethnicity was relatively high (86%), yet race was in an agreement between systems for only 53% of individuals; race data were often missing or unknown in one system, but not the other.

While insufficient to affect the post‐deduplication prevalence estimate for either condition, the selection method did influence the number of cases that we identified. The approach prioritizing diagnosis identified the largest number of cases (758 T1DM cases; 201 T2DM cases). Implementing alternate selection logic resulted in 7 to 10 fewer cases of T1DM and 5 to 11 fewer cases of T2DM, depending on the algorithm.

When restricting the analysis to ambulatory encounters, we observed a lower prevalence of T1DM (2.5 cases per 1000, 95% CI: 2.2‐2.8) than the prevalence including all encounter types (3.5 cases per 1000, 95% CI: 3.3‐3.7). There was no evidence that restricting to ambulatory encounters affected T2DM prevalence. As with the all‐encounter‐types analysis, in the ambulatory encounter‐only analysis, there was no observable change in prevalence after deduplication overall or for any demographic subgroups (results not displayed). We also identified fewer diabetes cases of either type in the ambulatory‐only analysis than in the all‐encounters analysis. We identified 399 fewer T1DM cases (53% of 758) and 58 fewer T2DM cases (29% of 201), depending on the choice of care setting (primarily) as well as the selection algorithm.

## 4 DISCUSSION

This study describes a process we designed to link and deduplicate individuals for prevalence estimate activities using a regional DDN. To our knowledge, this is one of the very few studies to report implementing HIPAA‐compliant IM across a DDN to generate deduplicated prevalence estimates.^24^

The process we designed and tested generated and stored a network‐wide identifier for use in distributed public health queries. The two‐step query process limited the amount of PII exchanged to a parsimonious limited data set. While we chose T1DM and T2DM in youth as the chronic conditions to test in the development of the algorithm, the deduplication method could be adapted to other chronic conditions including refinement for more prevalent or episodic conditions (eg, depression or substance use disorder). In addition, more refined case definitions for T1DM and T2DM could improve the accuracy of the reported prevalence estimates.

Importantly, unlike many population‐based surveys used for public health surveillance, DDN‐based prevalence estimates integrated with the LHS mindset provide a powerful approach to evaluating interventions in a given region. Prevalence is a metric that can help health systems, county health departments and others continuously learn how to best respond to pressing public health challenges, including but not limited to diabetes.

In this initial pilot test of our process, involving only two data partners, deduplication had no measurable effect on pediatric diabetes prevalence estimates. Very low disease prevalence estimates may have resulted in fewer opportunities for cross utilization. Analyses were limited to only two data partners; neither was a referral center for diabetes. The relatively small degree of overlap between the data partners was unexpected ‐ given referring relationships and geographic proximity ‐ and likely contributed to the null finding. Having selected just 1 year, there might have been greater utilization overlap if we extended the observation period. Approximately 4% of the 218 437 pediatric patients included in this pilot were represented in both systems during 2017 (n = 7628). The prevalence of T1DM and T2DM was higher in the duplicate population, but duplicates represented a very small share of the overall number of patients. Furthermore, individuals who were not assigned network identifier values (eg, missing critical matching variables) were excluded from this analysis. Our findings might have been different if the prevalence of diabetes or the degree of overlap differed considerably from the population that was included. Based on our work designing and evaluating this process, we identified several ways duplication bias might impact prevalence estimates.

**FIGURE 3:**
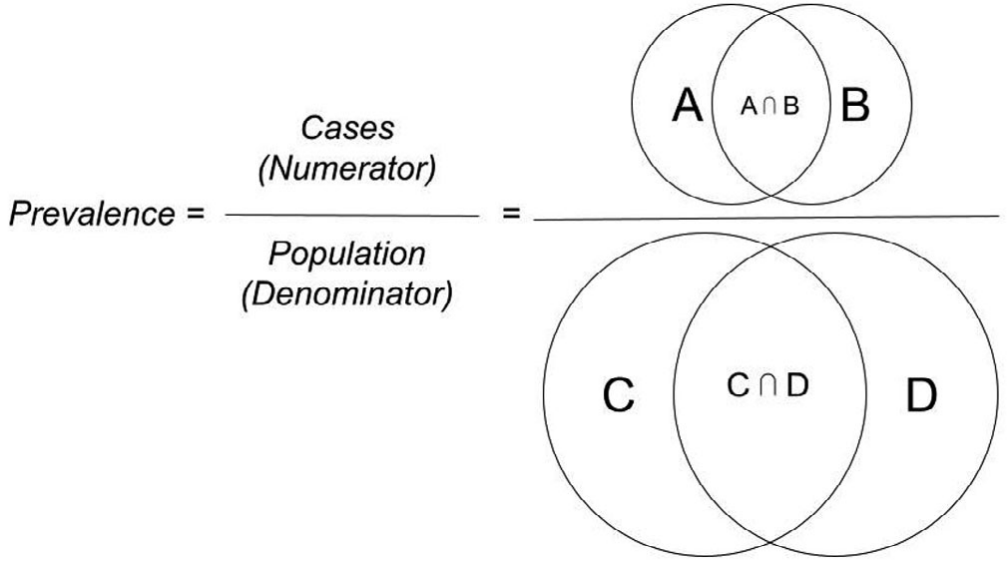
Opportunities for duplication bias when estimating disease prevalence from two data partners

*Duplication Bias and Potential Impact on Prevalence*: Duplication, if unaccounted for, has the potential to bias both prevalence estimate components (number of cases and people in the underlying population). Using simulated data, we represent potential overlap and bias among two hypothetical DDN data partners in Figure 3 and Table 5. Different degrees of overlap in the numerator and denominator have differing effects on deduplicated prevalence. Aggregated prevalence estimates are unbiased when data partners' populations are disjoint (ie, n(A ⋂ B) = 0, n(C ⋂ D) = 0) or when there is complete overlap (ie, C = D). Otherwise, bias is a matter of degree and can either inflate or deflate the prevalence estimate.

**TABLE 5:** Factors influencing prevalence (per 1000) under several scenarios of overlapping populations at two hypothetical data partners (DP1, DP2) with 1000 patients each

	DP1 prevalence per 1000	DP2 prevalence per 1000	Case overlap (n)	Population overlap (n)	Aggregated prevalence per 1000	Deduplicated prevalence per 1000
Set theory notation	n(A)/n(C) x 1000	n(B)/n(D) x 1000	n(A ⋂ B)	n(C ⋂ D)	(n(A) + n(B)) / (n(C) + n(D))	(n[A ⋃ B]) / (n[C ⋃ D])
Low prevalence: No overlap	5	5	0	0	5	5
Low prevalence: High population overlap	5	5	0	995	5	9.95
Low prevalence: Complete case overlap	5	5	5	5	5	2.51
Low prevalence: Complete overlap	5	5	5	1000	5	5
High prevalence: No overlap	500	500	0	0	500	500
High prevalence: High population overlap	500	500	0	500	500	666.6
High prevalence: Complete case overlap	500	500	500	500	500	333.3
High prevalence: Complete overlap	500	500	500	1000	500	500

*Note*: Aside from the top row, which uses set theory notation to represent the meaning of each column, each row illustrates how different combinations of conditions impact duplication bias in the prevalence estimate. Conditions that influence the degree of bias include: the prevalence of a condition (eg, low or high), the degree of overlap in the overall population [none, high (complete among non‐cases), or complete], and the degree of overlap in the case population (none or complete). Bias is introduced when overlap is disproportionate among cases and non‐cases.

When there is no overlap among cases and high overlap among non‐cases (eg, 100%), the aggregated prevalence is biased downward, with a greater downward bias in a lower prevalence scenario. When there is complete overlap among cases and no overlap among non‐cases, the aggregated prevalence is inflated ‐ likewise, to a greater degree when prevalence is lower.

The vast majority of DDNs, however, contain more than two data partners and have different sized populations ‐ both of which increase the complexity of estimating duplication bias effects. For example, calculating the deduplicated number of cases between two data partners (n(A) + n(B) − n(A ⋂ B)) is simpler than calculating the deduplicated number of cases involving three data partners (n(X) + n(Y) + n(Z) − n(X ⋂ Y) − n(X ⋂ Z) − n(Y ⋂ Z) + n(X ⋂ Y ⋂ Z)). Additional research leveraging set theory could help clarify the various ways in which prevalence could be biased as the number of data partners in a network increases. Furthermore, advanced simulations and analyses of real‐world data could help explore the impact of duplication bias under different scenarios (eg, different matching algorithms), which could help DDNs evaluate which deduplication processes would be worthwhile to implement. Regardless, in addition to the factors represented in Table 5 (higher/lower prevalence, overlap among cases, overlap among non‐cases), we hypothesize that the degree of duplication bias on prevalence estimation may also be influenced by other factors, including the relative sizes of the data partner patient populations (larger datasets may “overwhelm” data from smaller data partners), the sensitivity/specificity of the electronic phenotype, the accuracy of the patient matching algorithm, and whether the condition is chronic or episodic. Of course, more “traditional” sources of bias (eg, poor representativeness, poor data quality, disease misclassification) are still important to address. For example, patients had higher concordance on case status and gender than on ethnicity and race. Other studies have documented concerning levels of missing or misclassified race and ethnicity values in EHR data. One study reported that self‐recorded race and ethnicity differed from EHR data more than half of the time.^25^ Furthermore, if the matching algorithm has not been validated across population subgroups, the accuracy of the match might differ by race and ethnicity. The discordance we observed could be an artifact of misclassified race or ethnicity, differential matching by race or ethnicity, or a combination thereof.

*Electronic Phenotype*: At the time of this research more sophisticated case definitions for diabetes (eg, leveraging lab results) had not been tested or implemented in the CHORDS Network. Previous research has both underscored the importance of diabetes as a focus for EHR‐based surveillance, as well as the importance of accurate case definitions.^26^, ^27^, ^28^ This study focused on developing and describing DDN methods for deduplication using a relatively simple case definition to assess the impact of that method. No doubt, the accuracy of our reported prevalence estimates would improve with more sophisticated case definitions for type 1 and type 2 diabetes.

*Patient Matching*: Partnering with the HIE for patient matching provided several advantages. The HIE possessed the legal authority to receive and utilize PII to identify individuals across systems. The HIE also possessed the technical capacity to process millions of DDN records for IM. The patient‐matching component of our process, based on referential linkage methods, relies on a proprietary database. Evaluating the performance of the referential matching process, relative to other methods, was outside the scope of this study. That said, proprietary referential databases are created using publicly‐available data that are simply more available for adults than for children (who have had less time to generate such data). As such, the accuracy of referential matching for pediatric prevalence research requires further validation. Network identifiers generated with other matching methods,^29^, ^30^ when managed in the LINKAGE table, could be queried through the same two‐step process.

*Population Representativeness*: For both T1DM and T2DM, our local prevalence estimates were higher than reported national estimates,^4^ which could reflect a number of issues including local variation in Denver vs the country, the accuracy of the phenotype used (see above), the use of care‐seeking populations as denominators, non‐representativeness of these two systems' populations compared to the general population or a continued rise in prevalence of both types of diabetes among youth since previous estimates were reported.

## 5 CONCLUSION

We successfully implemented a process to deduplicate patients across health care systems to generate type 1 and type 2 diabetes prevalence estimates within a regional DDN. The process leveraged a master patient index at an HIE and limited exchange of protected health information to the minimum necessary. The process should be extensible to deduplicate data for specific patients from any number of data partners, and adaptable to other linkage methods, health conditions and cohort selection criteria. Results from this evaluation suggests several factors influence the duplication bias effect on cross‐institution prevalence estimates, including the relative size of patient populations, the representativeness of patients among participating data partners, organizational or patient self‐referral patterns, and shared patient populations. This process has informed how DDN prevalence estimates might be used to help learning health systems respond to public health challenges, track patients across a healthcare ecosystem and improve population health. Additional use cases (beyond diabetes) may refine our efforts to reduce bias and reveal other principles and insights.

## CONFLICT OF INTEREST

The authors have no conflicts of interest to declare.
